# School-based intervention to improve mental health, cognitive function, and academic performance in adolescents: a study protocol for a cluster randomised trial

**DOI:** 10.1186/s12889-026-26469-3

**Published:** 2026-02-04

**Authors:** Susanne Andermo, Björg Helgadóttir, Lisette Farias Vera, Örjan Ekblom, Gisela Nyberg

**Affiliations:** 1 Department of Neurobiology, Care Sciences and Society, Division of Nursing, Karolinska Institutet, Huddinge, 141 83 Sweden; 2 Department of Physical Activity and Health, The Swedish School of Sport and Health Sciences (GIH), Stockholm, 114 86 Sweden; 3 Department of Neurobiology, Care Sciences and Society, Division of Occupational Therapy, Karolinska Institutet, Huddinge, 141 83 Sweden; 4 Department of Global Public Health, Karolinska Institutet, Stockholm, 171 77 Sweden

**Keywords:** Physical activity, Adolescents, Mental health, Cognition, Academic performance, Schools

## Abstract

**Background:**

A majority of adolescents do not meet the recommended levels of physical activity, while reported levels of mental health problems are increasing, and socioeconomic disparities in academic performance are widening. Many schools are implementing physical activity in different forms, but there is inconclusive evidence on what types of interventions improve mental health, cognitive functions, and academic performance and how to implement such interventions. There is a critical need for integrated, feasible, and equitable interventions. The objective of this study is to develop an effective multi-component school-based intervention that will target both physical activity and homework support during an extended school day and evaluate its effects on mental health, cognitive function and academic performance.

**Methods:**

The study is designed as a cluster-randomised controlled trial with 54 schools and approximately 2,700 students in grade 8 (age 14–15). The intervention includes three weekly 60-minute sessions: (1) Different types of physical activities (2), Homework support with short activity breaks, and (3) Walking and listening to audiobooks. This study will evaluate both outcome effects and implementation process. The primary outcome is anxiety, assessed using the Spence Children’s Anxiety Scale (SCAS-S). Secondary outcomes include physical activity, sedentary time and behaviours measured by accelerometry and questionnaire, cognitive functions assessed by a computer-based test battery, mental health and sleep with questionnaires, and academic performance by grades. Process evaluation will include fidelity, dose, feasibility, acceptability and context, using structured documentation, interviews, focus groups, and observations.

The effectiveness of outcomes between groups will be assessed using mixed-effects regression analysis, adjusting for relevant covariates. Process data will be analysed using descriptive statistics and content, and thematic analysis.

**Discussion:**

This study addresses key knowledge gaps in school-based health promotion by integrating physical activity and homework support within the school structure. The results will yield insights into both effectiveness and implementation, informing future policy and practice in schools to promote health and facilitate students’ learning. The intervention targets youth in diverse socioeconomic contexts and is expected to contribute to reducing health and education inequalities.

**Trial registration:**

The trial was retrospectively registered on April 27, 2021. ISRCTN78666212.

**Supplementary Information:**

The online version contains supplementary material available at 10.1186/s12889-026-26469-3.

## Background

Being physically active during adolescence is associated with substantially higher mental and physical health in both the short- and long-term [1]. Despite this evidence, the majority of adolescents both in Sweden and internationally do not meet the physical activity recommendations [2, 3], and activity levels decrease with increasing age [4]. Critically, physical activity levels vary depending on socioeconomic background, where adolescents from families with low education show the lowest levels of physical activity, and lower academic performance [5]. Furthermore, more than half of children from the lowest socioeconomic backgrounds do not complete upper secondary education [6]. In parallel, cardiorespiratory fitness has declined over time [7], while the reported prevalence of mental health problems among Swedish adolescents has increased [8, 9]. Globally, it is estimated that 14% of 10–19-year-olds suffer from mental health conditions [10].

The prevalence of self-reported psychosomatic health problems has doubled between 1985 and 2018 [11] and 10–15% of children and adolescents have received health care for some form of mental illness [8]. In high-income countries in 2019, psychiatric conditions represented the most significant single contributor to the overall burden of disease among those under 20 years [12]. Anxiety is the mental health condition that increases most rapidly. Still, there is an evident lack of studies about anxiety in children and adolescents [9]. Evaluation of interventions and methods to promote mental well-being and prevent mental health problems is needed [9]. Mental health problems in early life are related to a higher risk of developing prolonged and more severe mental illnesses later, as well as poorer academic performance [8, 13, 14]. Adding to this, adolescents report decreased time in reading, and there is now a larger variation between students from different socioeconomic groups in reading comprehension compared to earlier education results [15].

Given such longitudinal trends in mental health, physical activity, fitness, and academic performance, it has been acknowledged that increasing levels of physical activity may represent an impactful intervention in adolescents. Physical activity has indeed been shown to be positively related to mental health, cognition, and academic performance in children and adolescents [16–18]. Adolescents who engage in more physical activity than their peers have reported positive effects on psychosocial outcomes and academic achievement [19]. Similar results have been reported in studies on twins, where a more active monozygotic twin reports higher mental health, compared to the less active twin, both cross-sectionally and longitudinally [20]. This renders an argument that at least some of the results from observational data can be regarded as causal, and gives the rationale for intervention studies in an applied setting. However, the evidence to date on the effects of school-based physical activity interventions on mental health, cognitive function and academic performance has not been consistent [17, 21, 22]. Of critical importance, school interventions have not been effective at actually increasing children’s physical activity, and there is a need for better implementation [23, 24]. More research is therefore needed to determine how school interventions, including physical activity, can be designed and implemented to be effective for increasing physical activity and improving mental health, cognitive function and academic performance in adolescents.

To better understand what type of strategies and implementation are effective for adolescents, more research is needed to understand how and what interventions and environmental factors can make an intervention more or less successful in their intended practice settings [25]. One of the challenges of school-based interventions is that teachers tend to identify time constraints as a major barrier to implementing physical activity programmes [26]. To address such barriers and enhance implementation, one important approach is to involve school staff and students in the planning process [27]. Such a research approach effectively generates knowledge that target groups find trustworthy, facilitating the transfer of evidence into practice [27].

Importantly, a change in the organisational structure by extending the school day represents a promising strategy for school-based programmes aiming to mitigate the impacts of social inequalities on adolescents’ health outcomes. Extending the school day additionally holds promise for improved implementation of homework support programmes, which tend to suffer from high attrition, sporadic attendance and mixed results relating to academic performance and behaviour [28, 29].

## Objective

The objective of this study is to develop an effective multi-component school-based intervention that will target both physical activity and homework support during an extended school day and evaluate its effects on mental health, cognitive function, as well as academic performance.

### Scientific research questions

1. What are the effects of a school-based programme including physical activity and homework support during an extended school day on mental and physical health, physical activity, cognitive function, and academic performance?
2. Are there any differences in the effects of the intervention in relation to gender, socioeconomic status, and neurodevelopmental conditions (e.g. ADHD, autism)?
3. How is the programme implemented with regard to fidelity, dose, feasibility, acceptability, and context? What are the barriers and facilitators identified in the implementation of the intervention?

## Methods

### Study design

This study will be carried out as a cluster-randomised parallel trial, with schools as the unit of randomisation and a wait-list control group. The list of schools that will participate will be computer-randomised either to intervention or control with a 1:1 ratio. The randomisation will be performed by a statistician in blocks of two and will be done separately for low and high socioeconomic status schools. The personnel recruiting the schools did not have access to the randomisation sequence. The protocol is reported in accordance with the SPIRIT (Standard Protocol Items: Recommendations for Interventional Trials) 2025 guidelines [30].

### Recruitment of schools, participants and setting

Schools from the Stockholm area within a 2–3 h drive from the Swedish School of Sport and Health Sciences and schools in the counties of Småland, located in the south of Sweden, will be invited by e-mail to the headteachers and vice headteachers. Schools with a sports profile and schools with a student population not speaking Swedish will be excluded.

Fifty-four schools with 2700 adolescents in grade eight, aged 14–15 years, will be included by the research group. Three waves are planned: the first in the school year of 2021–2022, the second in 2023–2024, and the third in 2024–2025. A one-year follow-up will be performed in the spring of 2025 and 2026.

### The intervention

A logic model of the intervention study, including the intervention components, process evaluation, and outcome evaluation, is described in Table 1.

**Table 1:** Logic model of the intervention study

Input	Intervention components	Materials	Implementation strategies	Mediators	Process outcomes	Outcome evaluation
Funding Expert support	Physical activities Homework support with short activity breaks Walking and audiobook	Physical activity inspirational material Digital audio book subscriptions	Workshops with school staff Parental meetings Research support	Competence Autonomy Relatedness	Fidelity Dose Feasibility Acceptability Context	Mental health Cognitive function Academic performance Physical activity Sedentary behaviour Health behaviours BMI

The development of the intervention has been directly informed by barriers and facilitators identified by students and teachers [31]. This information was collected through workshops and interviews during 2021–2022 and used to develop implementation strategies for this study.

### Intervention components

This multi-component intervention will be performed during an extended school day, 60 min three times per week, for six months and include three components described below. The intervention activities will be included as part of the mandatory school curriculum for all the students in the intervention schools.

1. Different types of physical activities (1 × 60 min per week). The physical activities will be chosen in collaboration with teachers and students, and will take place in both indoor school facilities and outdoor areas. Examples of activities are swimming, dancing, gym, circuit training and ball sports, to suit all interests.
2. Homework support with short activity breaks, 3–5 min every 30 min (1 × 60 min per week).
3. Walking and listening to an audiobook (1 × 60 min per week), in areas nearby the school.

To make the programme integrated and sustainable, the activities can be scheduled at any time during the school day and will be led by school staff.

### Theoretical framework

The intervention is based on evidence-based principles for supporting sustainable motivation and behaviour [32, 33]. The intervention is based on the Self-Determination Theory (SDT), which focuses on human motivation, particularly the three basic psychological needs of competence, autonomy, and relatedness. The intervention includes different types of physical activities, homework support with short activity breaks, and walking while listening to an audiobook. Participating in physical activities is expected to enhance physical skills and competence. The students will be able to choose physical activities that will promote their autonomy. The social and collaborative dimension of the activities also strengthens social bonds and the sense of relatedness. With a short activity break, homework support is expected to support the student’s sense of autonomy and competence. It will also create a supportive learning environment that enhances relatedness.

To optimise adherence, school staff will participate in workshops to improve their knowledge about health and learning and to plan and develop delivery plans for the intervention at the beginning of each wave. These workshops aim to enhance both staffs’ and students’ autonomy, competence, and relatedness. By aligning the intervention components with the principles of SDT, the programme creates a motivating and supportive environment for students, ultimately enhancing their overall well-being and academic performance.

### Implementation strategies

Several implementation strategies will be conducted to optimise adherence. These strategies include workshops, parental meetings, support from researchers, and inspirational material.

### Workshops with school staff

The school staff in the intervention schools will participate in workshops with the research team to plan how to design and implement the intervention in their schools. The workshops will focus on (1) feasible implementation strategies for the intervention components in each school and (2) how to enhance students’ and teachers’ involvement. An initial intervention delivery plan will be established at the end of the workshop. The school headteacher will be asked to provide facilities and extra time/hours for school staff to perform the intervention.

### Parental meetings

Parental meetings to inform about the study will be conducted in all intervention schools at the beginning of each school year. In these meetings, the researchers will give a short lecture about physical activity and health among adolescents and provide information about the study. The parents will have the opportunity to ask questions during the meeting.

Since the activities will be scheduled and mandatory for all students in intervention schools, the schools will also inform the parents of these activities at the beginning of the school term through their regular channels.

### Research support

Researchers will continuously visit all intervention schools to support the teachers with strategies on how to deliver the activities.

### Materials

All intervention schools will receive (1) Physical activity inspirational material, including digital material for the activity sessions, and activity cards for the activity breaks during the homework sessions, and (2) Digital audiobook subscriptions to use during the walking sessions.

### Control group

The control group will have unchanged school schedules and will be offered the intervention after the post-intervention measurements in the following school year.

### Outcome evaluation

This intervention study will evaluate both outcomes and the implementation process with quantitative and qualitative methods.

#### Data collection

Data for the outcome measures will be collected before and after the intervention. Baseline data will be collected in the autumn of 2021 (first wave), autumn 2023 (second wave), and autumn 2024 (third wave). Post-intervention data will be collected in the spring of 2022 (first wave), spring of 2024 (second wave), and spring of 2025 (third wave). One-year follow-up measures will be collected in the spring of 2025 and 2026. Data for the process evaluation will be collected continuously during the intervention (observations and documentation) during the school years: 2021–2022, 2023–2024, and 2024–2025. All measurement methods and routines have been tested with adolescents in the school setting.

Students in both the intervention and control groups will receive a gift card worth 150 SEK (≈ 15 €) as compensation after each measurement. Additionally, participants in the intervention group will receive audiobook subscriptions during the intervention while those in the control group will receive the same after the follow-up measurements.

#### Measurements

The measurements will be conducted by trained healthcare professionals and researchers who visit the schools and carry out the assessments before lunch on the test days. These measurements include questionnaires administered via a secure online survey tool, accelerometers to assess physical activity and sedentary time, height and weight measurements, and a cognitive test battery.

The parents, teachers or teachers’ assistants of physical activity sessions and headteachers also fill out online questionnaires. Reminders will be sent by e-mail and text messages up to three times.

##### Primary outcome

Anxiety will be assessed with the Spence Children’s Anxiety Scale Short Version (SCAS-S) questionnaire, including 19 items, which has been shown to be valid and reliable for this age group [34]. Anxiety will also be assessed with a subscale of the Measurement of Mental Health Among Adolescents and Young People at the Population Level (MMAPP) scale. The MMAPP comprises two subscales, depression and anxiety [35]. The instrument has been translated and culturally adapted for use in Sweden through a multi-step process involving direct engagement with adolescents and is currently undergoing validation [35].

##### Secondary outcomes

**Students**

Mental health will be self-reported in questionnaires. Health-related quality of life will be measured with the questionnaire Kidscreen-10 [36], stress with the single item stress question (SISQ) [37], psychosomatic health problems with the Psychosomatic Problem Scale (PSP), and self-esteem with the Rosenberg Self-Esteem Scale (RSES) [38].

Physical activity and sedentary time will be measured with accelerometry, which is an objective way of assessing detailed physical activity patterns [39]. For seven consecutive days, physical activity patterns will be measured with triaxial accelerometers (model GT3X+, Actigraph, LCC, Pensacola, USA). Participants will be instructed to wear the accelerometer on their right hip during all hours when awake except during activities involving water. The accelerometers will be sent back to the researchers in pre-paid envelopes. The software ActiLife Data Analysis, will be used to process the accelerometer data. Uniaxial (vertical axis) data will be saved in 5-second epochs. Non-wear time will be defined as 60 min of 0 counts, with no spike tolerance. A filter will be applied to distinguish school-time physical activity from leisure-time activity, based on school schedules for each class. A valid day is defined as having at least 500 min of activity registration per day, and a minimum of 3 days, including at least one weekend day, will be considered a valid week. School time will be considered valid if there are at least two valid weekdays. Sleep time will be based on the self-reported questions. The physical activity outcomes will be time spent in sedentary, light physical activity (LIPA), and moderate to vigorous intensity (MVPA). The counts from the accelerometer data will be categorised into minutes spent in: sedentary intensity (0–100 counts/minute), LIPA (101–2295 counts/minute), and MVPA (≥ 2296 counts per minute) [40].School time will be determined using the start and end times of each school day, as extracted from the school schedules. The weekly average of ≥ 60 min of MVPA per day will be used to reach the physical activity recommendation, based on both school and leisure time. In sensitivity analyses, activity intensity will be divided into 25 categories for high resolution analysis. In these analyses, the innate low-pass filter at 1.63 Hz will be removed and replaced with a 4 Hz cut-off for better validity [41].

Cognitive function will be assessed using a computer-based test battery measuring working memory (letter updating), episodic memory (word recall), and perceptual speed (number comparison). Using three different domains of cognitive function and multiple trials allows for a broader understanding of cognitive function and increases the reliability of the test while keeping participant burden to a minimum. This design also allows for the construction of a latent variable for cognitive function. A previous study has shown a version of this test battery to be a valid and robust measure of these cognitive abilities in an older population [42].

Academic performance in Swedish, English, and mathematics will be measured by grades and standardised Swedish national tests collected from the Statistics Sweden (SCB) register (grade 9) and directly from the schools (grades 7 and 8).

Body mass (kg) and height (cm) will be measured in duplicates using standardised procedures with SECA instruments. Body Mass Index (BMI) will be calculated as body mass (kg) divided by height (m) squared. Overweight and obesity will be defined according to the International Obesity Task Force standard deviation score (BMI sds), computed according to a Swedish reference standard [43].

Health behaviours will self-reported in a questionnaire. Physical activity and sedentary behaviours include questions about transport to and from school, physical education attendance, participation in organised sports/activities, and screen time use. Dietary intake will be assessed by self-report in a questionnaire, based on a composite score of indicator foods, including healthy foods (fruit and vegetables), unhealthy foods (sweets and ice cream), and sugar-sweetened beverages.

Sleep will be measured and expressed as total sleep time and efficiency using a questionnaire where the participants report when they usually go to bed and wake up.

Motivation to physical activity will be measured using an adapted version of the Behavioural Regulation In Exercise Questionnaire (BREQ) [32].

Functional disabilities will be self-reported using the Modified Child Functioning Module [44].

Background characteristics: Parental educational level will be collected from the SCB register as an indicator of socioeconomic status. The highest level of education attained in the household will be used and dichotomised into low education (≤ 12 years) and high education (> 12 years). Parental country of birth will be self-reported in a questionnaire. Information about school type (independent or public) will be collected from the National Agency for Education.

**Headteachers**

Information about the school environment in relation to opportunities for physical activity and current health-related policies within each school will be self-reported by headteachers in questionnaires.

**Parents**

Information about family background, lifestyle, the child’s physical activity, health, and possible medical diagnoses or disabilities, including neurodevelopmental disorders, will be self-reported by parents in questionnaires.

### Process evaluation

The process evaluation includes exploring the implementation of the intervention, with a focus on fidelity, dose, feasibility, acceptability, and contextual factors [45]. A combination of qualitative and quantitative methods will be used.

#### Fidelity and dose

Fidelity will be assessed through structured documentation by the teachers involved in delivering the intervention. Teachers will be asked to document all performed activities. For the physical activity sessions, the teachers will document the type and intensity of the activities. In addition, the teachers will document any adaptations made to the intervention protocol and the reasons for these adaptations. Implementation dose will be assessed as the dose delivered (the number of sessions) and the dose received (student participation), provided by teachers.

#### Feasibility, acceptability and context

The feasibility, acceptability and context will be explored through qualitative focus groups [46, 47] and/or interviews [48] with both students and teachers and through observations [49]. Students will be invited to focus groups (*n* = 20–30), with 6–8 participants in each focus group. A selection of students from each school will be invited to include a balanced gender representation, with a variation of engagement in the intervention activities. Teachers, headteachers and/or vice headteacher will be invited to interviews (*n* = 40) and/or focus groups. The selection will be based on male and female teachers, representing a variation of engagement in the delivery of the intervention and to represent various subjects. Interview guides focusing on the participants’ and teachers´ experiences of the intervention, including its different components, contextual factors, perceived facilitators and barriers, gender aspects, student needs, and potential influence of other aspects on intervention delivery and outcome, will be used.

In addition to interviews and focus groups, researchers will conduct observations during the intervention activities at all participating schools [49]. Researchers will conduct observations during a selection of intervention activities in all participating schools to deepen the understanding of how the intervention is implemented in practice. These observations will be systematically documented, focusing on adherence to the planned content, level of student engagement, teacher-student interaction, and any contextual influences that may have affected delivery. Field notes will be taken during or immediately after each observation, using a structured template to ensure consistency across settings.

### Sample size calculation

To obtain an effect size of intervention Cohen’s d = 0.35 in anxiety (based on a meta-analysis [18]) in a population with the average correlation between cluster of 0.02, a variance of 5 points in the primary outcome SCAS-S, a two-tailed significance test (α = 0.05), and a power of 80%, it is required to include at least 27 schools in the intervention group and 27 schools in the control group with 50 students from each school. Anxiety will be assessed with the questionnaire Spence Children’s Anxiety Scale Short Version (SCAS-S) [34].

### Data management

The participants will be assigned a unique ID number and data will be de-personalised. The code linking the ID number to personal information will be stored separately on a secure server in a password-protected document. Original paper records will be securely stored at the Swedish School of Sport and Health Sciences to allow for quality control of electronic data entry. Data from the accelerometers will be transferred to electronic files, securely stored, and then deleted from the devices. All project data will be securely stored in password-protected electronic files on a server at the Swedish School of Sport and Health Sciences to prevent unauthorised access. Raw data files will be kept separate from those used in active analysis, and no files used in statistical analyses will contain any personally identifiable information. To reduce the risk of data loss, systematic backups will be performed throughout the data collection process, and data will be stored on servers that are backed up daily. Access to the data will be restricted to researchers directly involved in data entry or analysis. No data monitoring committee (DMC) will be established due to the trial’s low risk. Adverse effects will be monitored by the research group. No specific provisions for ancillary or post-trial care are planned. However, participants who suffer harm from trial participation will be covered according to the institution’s standard insurance.

### Data analysis

Baseline differences in continuous demographic variables between the intervention and control groups will be presented using means and standard deviations (SD) for normally distributed data and compared using independent t-tests. For non-normally distributed data, medians and ranges will be reported, with analyses conducted using non-parametric tests. Categorical variables will be described as proportions, and group differences will be examined using chi-square tests. The effects of the intervention on primary and secondary outcomes will be analysed through mixed-effects regression models with two hierarchical levels (individual and school), using post-intervention data and following the intention-to-treat approach. Initially, a crude model will be tested for each outcome at post-intervention, including group allocation as a predictor and adjusting for baseline values of the corresponding outcome. In a second model, gender and parental education will be added to the model. Interactions between group and gender, or group and parental education, will be tested and, if significant, analysed using stratified models All participants with baseline measurements will be included, and missing data will be addressed using multiple imputation The potential impact of missing data on the results will be evaluated through sensitivity analyses. Additionally, the analysis will be performed per protocol, including only those participants with high fidelity to the intervention components. All analyses will be performed blinded to group allocation. The data analysis plan was submitted as part of the trial registration (ISRCTN78666212).

All focus groups and interviews will be audio-recorded, transcribed verbatim, and analysed using qualitative analysis, including content [50–52]- and thematic analysis [53, 54]. The documented data on dose and fidelity will be analysed with descriptive statistics.

## Discussion

Increasing health inequalities, declining physical activity and fitness, and an increase in mental health problems among adolescents require in-depth knowledge and new strategies for preventive work. This theory-driven, multi-component school-based intervention addresses a critical knowledge gap in how to effectively promote mental well-being, physical activity, and academic performance among adolescents. Although schools are increasingly recognised as key settings for health promotion, there is still limited evidence on how to design and implement integrated programmes that are both evidence-based and feasible within existing school structures. By integrating physical activity and homework support within an extended school day, this novel project has the potential to sustainably improve adolescent health and learning.

The intervention combines physical activity and homework support, scheduled within an extended school day, and is designed to be integrated into existing school structures and routines. By taking place during regular school hours and being delivered by school staff, the programme may help overcome common barriers such as time constraints and limited capacity for additional initiatives. This design is expected to enhance the feasibility of implementation and increase the likelihood of long-term sustainability, thereby contributing to improvements in adolescent health and academic achievement. To ensure fidelity and quality, school staff receive ongoing support throughout the intervention period.

A thorough process evaluation is a crucial part of intervention evaluation, as it provides a contextual understanding of both how the intervention is delivered and the resulting outcomes. Understanding each component and the delivery process is essential to clarify the mechanisms and contextual factors influencing the effectiveness of the intervention. Therefore, there is a need to examine all aspects of a programme, especially in complex multi-component interventions, as we have intended to do in this intervention.

One strength of this study is its theoretical foundation, linking intervention components to intended outcomes, combined with a strong study design. One of the weaknesses is that schools are expected to implement the programme using their own resources, including staff time and facilities, which may lead to challenges. By extending the school-day, the risk is minimised that teachers and school-staff experience increased stress in normal teaching and work. In many countries, Sweden included, there are reports on high workload among school staff and teachers [55]. Adding intervention activities during the limited, mandatory school hours increases the risk of an even higher perceived workload and thereby limits feasibility and sustainability.

The findings from this novel project will guide the development of evidence-based advice and tools for schools to design curricula and develop sustainable and effective strategies aimed at improving mental health, cognitive function, and academic performance among students. The results from the project will be reported in scientific articles as well as summarised in short reports to be shared with the participating schools. Well-defined, evidence-based advice will ensure that resources are directed toward activities proven to be effective. In addition, the results are expected to contribute valuable knowledge with the potential to reduce health inequalities and social costs, especially by providing equitable opportunities for adolescents from socioeconomically disadvantaged backgrounds.

## Supplementary Information

Supplementary Material 1.

## Data Availability

Data sharing is not applicable, as no datasets have been generated.

## Abbreviations

BMI: Body Mass Index
BREQ: Behavioural Regulation in Exercise Questionnaire
ID: Identification number
LIPA: Light Intensity Physical Activity
MMAPP: Measurement of Mental Health Among Adolescents and Young People at the Population Level
MVPA: Moderate to Vigorous Physical Activity
PA: Physical Activity
PSP: Psychosomatic Problem Scale
RSES: Rosenberg Self–Esteem Scale
SCAS: S–Spence Children’s Anxiety Scale Short Version
SCB: Statistics Sweden
SISQ: Single Item Stress Question
SDT: Self–Determination Theory
